# Hypothermic Oxygenated Machine Perfusion of Extended Criteria Kidney Allografts from Brain Dead Donors: Protocol for a Prospective Pilot Study

**DOI:** 10.2196/14622

**Published:** 2019-10-14

**Authors:** Franziska Alexandra Meister, Zoltan Czigany, Jan Bednarsch, Jörg Böcker, Iakovos Amygdalos, Daniel Antonio Morales Santana, Katharina Rietzler, Marcus Moeller, René Tolba, Peter Boor, Wilko Rohlfs, Ulf Peter Neumann, Georg Lurje

**Affiliations:** 1 Department of Surgery and Transplantation University Hospital, Rheinisch-Westfälische Technische Hochschule Aachen Aachen Germany; 2 Division of Nephrology, Department of Medicine II University Hospital, Rheinisch-Westfälische Technische Hochschule Aachen Aachen Germany; 3 Institute for Laboratory Animal Science and Experimental Surgery University Hospital, Rheinisch-Westfälische Technische Hochschule Aachen Aachen Germany; 4 Institute of Pathology University Hospital, Rheinisch-Westfälische Technische Hochschule Aachen Aachen Germany; 5 Institute of Heat and Mass Transfer Rheinisch-Westfälische Technische Hochschule Aachen Aachen Germany

**Keywords:** hypothermic oxygenated machine perfusion, donation after brain death, extended criteria donor, kidney transplantation, kidney transplant, organ donation

## Abstract

**Background:**

Kidney transplantation is the only curative treatment option for end-stage renal disease. The unavailability of adequate organs for transplantation has resulted in a substantial organ shortage. As such, kidney donor allografts that would have previously been deemed unsuitable for transplantation have become an essential organ pool of extended criteria donor allografts that are now routinely being transplanted on a global scale. However, these extended criteria donor allografts are associated with significant graft-related complications. As a result, hypothermic oxygenated machine perfusion (HOPE) has emerged as a powerful, novel technique in organ preservation, and it has recently been tested in preclinical trials in kidney transplantation. In addition, HOPE has already provided promising results in a few clinical series of liver transplantations where the liver was donated after cardiac death.

**Objective:**

The present trial is an investigator-initiated prospective pilot study on the effects of HOPE on extended criteria donor allografts donated after brain death and used in kidney transplantation.

**Methods:**

A total of 15 kidney allografts with defined inclusion/exclusion criteria will be submitted to two hours of HOPE via the renal artery before implantation, and are going to be compared to a case-matched group of 30 patients (1:2 matching) who had kidneys transplanted after conventional cold storage. Primary (posttransplant dialysis within 7 days) and secondary (postoperative complications, early graft function, duration of hospital and intensive care unit stay, and six-month graft survival) endpoints will be analyzed within a six-month follow-up period. The extent of ischemia-reperfusion injury will be assessed using kidney tissue, perfusate, and serum samples taken during the perioperative phase of kidney transplantation

**Results:**

The results of this trial are expected in the first quarter of 2020 and will be presented at national and international scientific meetings and published in international peer-reviewed medical journals. The trial was funded in the third quarter of 2017 and patient enrollment is currently ongoing.

**Conclusions:**

This prospective study is designed to explore the effects of HOPE on extended criteria donor kidney allografts donated after brain death. The present report represents the preresults phase.

**Trial Registration:**

Clinicaltrials.gov NCT03378817; https://clinicaltrials.gov/ct2/show/NCT03378817

## Introduction

Since Joseph Murray’s pioneering efforts in 1954, allogenic kidney transplantation has evolved as the standard treatment for end-stage renal disease [1]. In 2018, approximately 8000 patients were waiting for kidney transplant in Germany, but only 2005 transplant procedures using postmortem organs were performed due to organ scarcity [2]. Several strategies for donor pool expansion are being pursued concurrently. These include the use of old donors, living donors, and extended criteria donor (ECD) allografts for kidney transplant. However, ECD-allografts exhibit poor tolerance to ischemia-reperfusion injury (IRI), an important cause of kidney damage [3,4]. As such, IRI is usually the underlying cause of graft dysfunction in ECD kidney transplant [5]. Primary graft nonfunction, leading to graft loss and retransplantation, as well as delayed graft function (DGF) with the need for posttransplant dialysis are the most frequent clinical manifestations of this pathology [5].

Dynamic organ preservation approaches, such as machine perfusion, hold promise for improving organ preservation and resuscitation of marginal donor allografts [6-9]. Although tissue oxygen consumption is markedly decreased at 4-10°C, there is still relevant metabolism at this temperature. Oxygen during machine perfusion is the key ingredient in the protective responses of hypothermic oxygenated machine perfusion (HOPE), with organ reconditioning effectively increasing the cellular energy balance via various mitochondrial pathways [10]. Over time, the addition of oxygen during machine perfusion emerged as a powerful novel tool in organ preservation [11,12]. HOPE is an easy to implement, short-term, end-ischemic reconditioning concept, as the organ perfusion is performed in the transplant center shortly before the actual implantation [11,13]. Ischemia-reperfusion injury occurs upon revascularization of the kidney allograft. Decrease of oxidative metabolism and depletion of adenosine triphosphate (ATP) are the results of the lack of oxygen during ischemia. Reintroduction of oxygen to the ischemic allograft results in massive reactive oxygen species production and release, leading to cellular damage. The mechanisms behind IRI are multifactorial, and ways to minimize its detrimental effects are still the subject of intense debate [14].

**Figure 1:**
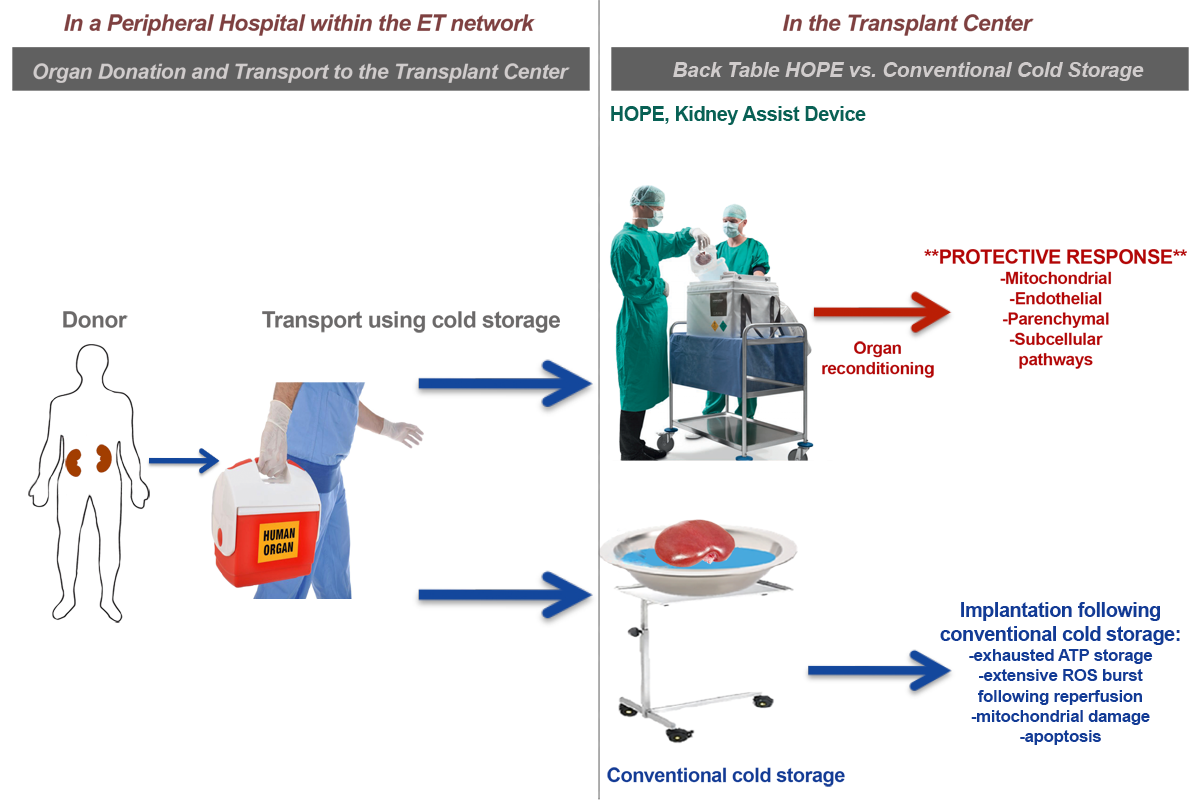
Hypothermic oxygenated machine perfusion. The donor organ is explanted and transported to the transplant center stored in a box of ice. In the transplant center, the kidney is connected to the Kidney Transport Assist device and HOPE is performed. It is assumed HOPE leads to less oxidative stress, decreased cell death and enhanced energy reserves. Adapted from Czigany et al [&lt;xref ref-type="bibr" rid="ref12"&gt;12&lt;/xref&gt;]. ET: Eurotransplant, HOPE: hypothermic oxygenated machine perfusion; ATP: adenosine triphosphate; ROS: reactive oxygen species.

In the preclinical setting, the positive effects of HOPE have recently been demonstrated through a reduction in the incidence of tubular damage, macrophage activation, and functional optimization of cellular energy-status in vitro and in vivo [8,9,11]. There are several hypotheses regarding the underlying mechanisms of HOPE-induced organ protection. According to these hypotheses, HOPE presumably exerts its positive effects by: (1) modulating cellular metabolism (energy balance, mitochondrial respiration, IRI); (2) stimulating the vascular endothelial layer; and (3) triggering various subcellular protective pathways (Figure 1) [15]. Investigating the effects of HOPE in a rat model, Kron et al recently demonstrated its beneficial effects on the immune response. Less cytokine release and less T-cell and macrophage activation were measured in the animals receiving the HOPE-treated kidneys [16]. Accordingly, decreased immune response may be one of the key underlying mechanisms of HOPE.

The aim of this prospective cohort study is to investigate the effects of HOPE on ECD kidney allografts donated after brain death. This study utilizes a comprehensive sample collection protocol to assess the subcellular mechanisms of HOPE in the clinical setting.

## Methods

### Study Type and Eligibility

**Figure 2:**
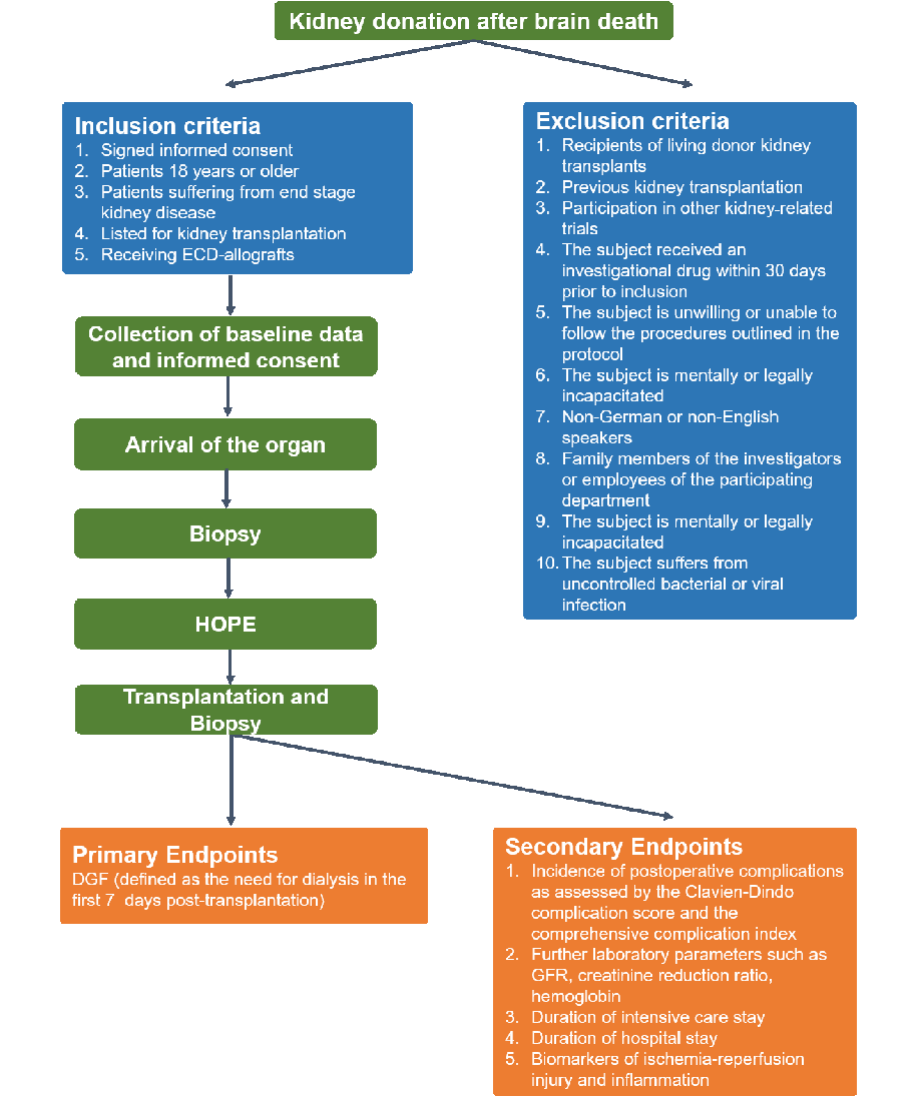
Study flow chart. ECD: extended criteria donor; HOPE: hypothermic oxygenated machine perfusion; DGF: delayed graft function; GFR: glomerular filtration rate.

The present trial is an investigator-initiated pilot study on the effects of HOPE on ECD-allografts in donated after brain death kidney transplants. Figure 2 summarizes the trial design.

Patients older than 18 years of age, suffering from end-stage renal disease (ESRD), listed for kidney transplant, and receiving ECD organs at the Department of Surgery and Transplantation, University Hospital Rheinisch-Westfälische Technische Hochschule (RWTH) Aachen, Aachen, Germany, are eligible for the study. Informed consent was obtained from all subjects participating in the trial by a qualified member of the study team. Inclusion and exclusion criteria are listed in Textbox 1.

**Study inclusion and exclusion criteria.**

*Inclusion Criteria:*

- Signed informed consent
- 18 years or older
- Suffering from ESRD (end-stage renal disease)
- Listed for kidney transplant
- Receiving ECD (extended criteria donor)-allografts

*Exclusion criteria:*

- Recipients of living donor kidney transplants
- Previous solid organ transplantation
- Combined procedures (eg, kidney-liver transplantation)
- Participation in other kidney-related trials
- The subject received an investigational drug within 30 days prior to inclusion
- The subject is unwilling or unable to follow the procedures outlined in the protocol
- The subject is mentally or legally incapacitated
- Non-German or non-English speakers
- Family members of the investigators or employees of the participating department
- The subject suffers from uncontrolled bacterial or viral infection

### Organ Procurement and Definition of Extended Criteria Donor Criteria

ECD kidney allografts will be retrieved by local procurement teams with the Eurotransplant network. Following cross-clamping (*in situ* flushing of the abdominal organs and the beginning of cold ischemia time), allografts will be removed and transported in a standardized fashion on packed ice. Required data regarding the donor and the organ will be collected and will be transferred to designated case report files (CRFs).

As per previous studies [4,17] deceased donors must be ≥60 years old, but it is possible for those aged between 50-59 years old to be donors if they have at least two of the following conditions: (1) cerebrovascular cause of death; (2) serum creatinine greater than 1.5 mg/dL (132.6 µmol/L); or (3) history of arterial hypertension.

### Hypothermic Oxygenated Machine Perfusion Versus Historic Cold Storage Group

Primary and secondary outcomes of patients transplanted with the HOPE-treated kidneys will be compared to a case-matched group of 30 patients (1:2 matching) transplanted with ECD organs after conventional cold storage, using identical surgical techniques and perioperative treatment at the Department of Surgery and Transplantation, University Hospital RWTH Aachen. The present trial comprises two groups, a perfusion (Group 1, HOPE) and a control conventional cold storage group (Group 2, Historic). Patients on the waiting list for kidney transplant with proven written consent will be recruited. HOPE will be applied to the allograft in the operating room after regular organ procurement, transport, and back-table preparation (Figure 1).

We adapted a protocol based on clinical studies with nonoxygenated hypothermic machine perfusion, and on preclinical experience with HOPE [11,18,19]. HOPE will be applied through the renal artery for at least two hours, with a perfusion pressure of 25 mmHg and a perfusion rate of 50-200ml/min using 1 L of recirculated perfusate at 0-4°C. The perfusate will be oxygenated using medical grade oxygen (O^2^), with a partial pressure of oxygen (pO^2^) of 60-80 kilopascals (kPa), by an oxygenator included as a disposable part of the setup. Perfusion parameters registered by the device will be stored automatically and evaluated for possible patterns. Immediately prior to perfusion, grafts will be flushed with perfusate solution to wash out the residual histidine-tryptophan-ketoglutarate (HTK) solution. Storage, management, and use of the medical products will be carried out according to the manufacturer’s guidelines.

### Matching Criteria

Donor urine output over the last 24 hours before retrieval, cold ischemic time (CIT), and the recipients Charlson comorbidity index [20] have been selected as matching criteria. Matching will be performed using a propensity score [21].

### Kidney Transplantation

Kidney grafts will be implanted heterotopically to the iliac fossa, as per our institutional protocol. The renal vein is anastomosed first, end-to-side to the external iliac vein, followed by renal artery anastomosis to the external iliac artery in the same fashion. After reperfusion of the kidney, the ureter is implanted into the bladder, according to Lich-Gregoir, and the uretero-cystostomy is stented with a 6-French double-J stent.

### Sample Collection and Storage

**Figure 3:**
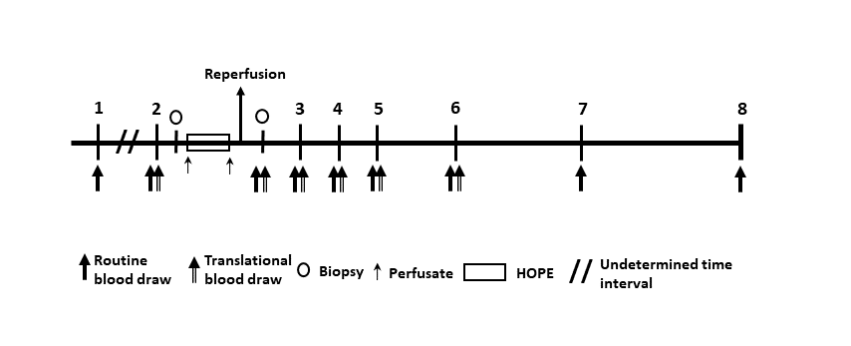
Interventions and study visits. Numbers represent the single study visit: Visit 1: screening, enrollment; Visit 2: admission; Visits 3, 4, and 5: postoperative days 1, 2, and 3; Visit 6: seventh postoperative day; Visit 7: discharge; Visit 8: 6 months follow-up; final visit. HOPE: hypothermic oxygenated machine perfusion.

Ischemia reperfusion injury will be assessed using kidney tissue samples taken upon arrival of the organ (before HOPE) and at the end of implantation after reperfusion, but before closure of the abdomen, to evaluate the extent of IRI (Figure 3). In total, two biopsies using BARD MONOPTY Disposable Core Biopsy Instrument (Bard Biopsy Systems Inc, Tempe, Arizona) will be harvested from the ECD kidney allograft and used for translational research. During machine perfusion, perfusate samples will be collected every 20 minutes. Blood samples are taken as part of the daily routine during the perioperative and postoperative course of kidney transplant (Figure 3). Blood parameters of kidney function (ie, creatinine, urea, estimated glomerular filtration rate [eGFR]) will be monitored. An additional 20 mL of blood will be drawn on postoperative days 1, 2, 3 and 7 and will be used for translational research (Figure 3). All kidney tissue, serum, and perfusate will be directly snap-frozen in liquid nitrogen (−80°C) and stored for 6 months after completion of the trial. To assess the subcellular mechanisms of HOPE in the clinical setting, various parameters, including markers of kidney injury (creatinine and neutrophil gelatinase-associated lipocalin (NGAL) in plasma samples), tissue ATP concentration, inflammatory mediators (eg, tumor necrosis factor alpha, interleukin-6, macrophage migration inhibitory factor, interleukin-10, monocyte chemoattractant protein-1), and biomarkers of oxidative damage (eg, 8-hydroxy-2'-deoxyguanosine), will be analyzed [11,12].

### Immunosuppression and Postoperative Care

All patients will be treated according to our institutional protocol. Aside from HOPE for the donor kidneys, all procedures, including peri- and intraoperative management, are conducted in accordance with standard surgical kidney transplant management. The applied immunosuppressive regimen is based on intraoperative induction therapy with intravenous methylprednisolone and either basiliximab or thymoglobulin followed by corresponding oral doses of prednisolone, tacrolimus, and mycophenolate mofetil.

### Study Endpoints

The primary endpoint is DGF, which is defined as the need for dialysis in the first 7-days posttransplantation. The secondary endpoints of the study include: (1) duration of DGF (defined as the period between kidney transplant and last dialysis in days) and functional DGF (defined as <10% fall in serum creatinine for 3 consecutive days in the first week posttransplantation); (2) creatinine reduction ratio day 2 ([creatinine day 1−creatinine day 2]/creatinine day 1) and Creatinine reduction ratio day 5 ([pretransplant creatinine−creatinine day 5]/pretransplant creatinine); (3) incidence of postoperative complications, as assessed by the Clavien-Dindo complication score and the comprehensive complication index [22]; (4) duration of intensive care and hospital stay; (5) recipient and graft survival after six months; (6) renal function (assessed by serum creatinine and eGFR) at one-, three- and six-months post-transplant; and (7) basic and translational research, which involves assessing biomarkers from routinely harvested kidney tissue and serum following implantation to obtain information on kidney injury, extent of oxidative damage, redox- and energetic homeostasis of the implanted donor kidney.

### Study Population

To the best of our knowledge, aside from a sole case of HOPE-treated dual kidney transplant reported recently, no experience of HOPE in human donation after brain death kidney transplant has been published yet [23]. In the present pilot study, 15 patients (N=15) will be treated with HOPE to gain first prospective clinical knowledge. HOPE-treated patients will be compared to a case matched group of 30 patients (N=30).

### Data Collection and Statistics

Patients will be given consent forms while in our outpatient clinic. Data will be obtained using study specific CRFs completed by the members of the study team. Subjects will be informed about data protection, including pseudonymization. Encoded data will only be provided to authorized persons (ie, authorized study staff, authorities, institutional review board). The study will be prematurely terminated for any individual subject in case of study-related complications or if the subject withdraws informed consent. Parametric and nonparametric tests will be done according to the normality of the data distribution. Two-way analysis of variance will be used for the time-course analysis of laboratory parameters. The log-rank test will be used for comparisons between Kaplan-Meier curves of six-month graft survival. Statistical significance will be defined as *P*<.05.

### Safety Considerations

Exclusively certified medical products will be used. Blood sampling and tissue biopsies during the transplant procedure will be performed according to clinical standards, preventing any relevant study-related risks or additional burden on the subjects. An interim analysis will be performed as soon as 10 patients are HOPE-treated. The trial will be terminated immediately if the following criteria is fulfilled: significantly higher DGF rate (*P*<.05 using the Chi-squared test) in the HOPE group compared to the matched conventional cold storage group (efficacy).

### Ethics

The present trial will be carried out in compliance with the current version of the Declaration of Helsinki, good clinical practice guidelines (International Conference on Harmonization-Good Clinical Practice [ICH-GCP]), and all national legal and regulatory requirements. The institutional review board of the University RWTH Aachen has approved the study protocol, including consent form and patient information leaflet (EK 184/17). Members of the study team have completed a course in good clinical practice as certified by the German Medical Chamber.

### Study Group

The study group of the present trial comprises the trial sponsor (GL, UPN) and the principal investigator (GL) of the University Hospital RWTH Aachen

### Ischemia-Reperfusion Injury and Inflammation

IRI is an inevitable event in organ transplantation. Reduction of oxygen delivery because of blood flow interruption leads to anaerobic metabolism and the production of oxygen-free radicals and oxidative stress as a respiratory burst at the onset of reperfusion. IRI plays a key role in DGF and primary graft nonfunction following kidney transplantation, depending on initial organ quality [8,9,24,25]. To detect the effects of HOPE on IRI, the kidney tissue, blood, and perfusate samples obtained in this study will be used to quantify several innovative parameters of inflammation (eg, interleukins, tumor necrosis factor-alpha), kidney injury (Cytatin-C, NGAL, etc) energy- (ATP levels) [11,26], and redox-threshold (Hemoxygenase-1, Malondialdehyde) [12,27]. Luminometry, Spectrophotometry, Luminex-assay, Enzyme-linked immunosorbent assay (ELISA), Reverse transcriptase polymerase chain reaction (RT-PCR) and Western blot will be used for these analyses. Proteomics and metabolomics analysis will be performed on kidney tissue samples to potentially identify early mediators of HOPE-mediated organ protection.

## Results

Results of this trial will be presented at national and international scientific meetings and published in international peer-reviewed medical journals. The trial was funded in the third quarter of 2017. Patient enrollment is currently ongoing, and the first results are expected in the first quarter of 2020.

## Discussion

Allogenic kidney transplantation has evolved as the mainstay of treatment for end-stage renal disease [3,28]. The decreasing availability of quality organs for transplantation has resulted in substantial organ shortage, and as such kidney donor allografts that would have previously been deemed unsuitable for transplantation have become an essential organ pool of ECD-allografts that are now routinely being transplanted on a global scale. However, the use of ECD allografts is associated with IRI and a higher incidence of primary graft nonfunction or DGF [5]. The restoration of blood flow after cold and warm ischemia leads to paradox via massive release of reactive oxygen species, cytokines, chemokines, and activation of leukocytes [29]. Although machine perfusion in kidney transplantation was explored in the 1970’s, it has not been widely used over the last few decades. Due to technical improvements and the addition of oxygen during machine perfusion, HOPE has evolved into a promising novel tool in organ preservation [11,12]. The superiority of HOPE over nonoxygenated cold machine perfusion and conventional cold storage was recently demonstrated in some preclinical experiments as well as in clinical trials using HOPE in donation after cardiac death liver transplantation [11,12,15]. A recent kidney transplantation rodent model in vivo study by Kron et al compared conventional cold storage, normothermic oxygenated blood perfusion, HOPE, and hypothermic deoxygenated perfusion using nitrogenated perfusate, and demonstrated superior effects of HOPE on macrophage activation, endothelium activation, tubular damage, and graft function as compared to other preservation methods [11].

We identified four active clinical trials exploring the effects of HOPE in kidney transplantation. In a pilot study by Ravaoili et al, 20 subjects were recruited to receive either HOPE-treated ECD-kidney or ECD-liver allografts. Whether the organs used were donated after cardiac death or donated after brain death remains unclear (NCT03031067; Table 1). One trial (NCT02621281) is a large multi-center RCT investigating HOPE in donated after cardiac death kidney transplantation, including ECD and nonECD allografts in a large Chinese cohort. Two relevant trials of the Consortium for Organ Preservation in Europe are recruiting in a large collective of patients. The Cold oxygenated machine preservation of aged renal donation after cardiovascular death transplants (COMPARE) trial (ISRCTN32967929), initiated at the University Hospital Leuven, Belgium, included a total of 102 transplanted donated after cardiac death kidney pairs that are being used to discover whether continuous oxygenated machine perfusion is superior to nonoxygenated machine perfusion. The COMPARE trial has a considerably different design compared to our study, with a logistically complex perfusion concept involving the transport of the machines to the retrieval center and applying continuous HOPE. Preimplantation oxygenated hypothermic machine perfusion reconditioning after cold storage (COPE-POMP) (ISRCTN63852508), initiated from the University Hospital of Essen, Germany, is currently ongoing and involves randomizing ECD allografts to end-ischemic preimplantation HOPE or to conventional cold storage.

Although we have designed our trial carefully, nonrandomization of the study groups and nonblinding of the transplant team, as well as matching with a historical group, are limitations of the present study. Nevertheless, conclusive clinical data about end-ischemic HOPE in human kidney transplantation are still missing and larger multi-center studies on this topic are currently underway. Therefore, it seems plausible to conduct this trial as a pilot study with a focus on short-term outcomes as well as translational research aspects of HOPE in ECD-kidney transplantation.

The present trial, nevertheless, possesses some specific strengths in that it focuses on patients solely receiving ECD-allografts, a population in which we anticipate the best cost/benefit ratio from the utilization of HOPE. Kidney transplant outcomes stem from a combination of various factors. Our matching criteria have been carefully selected based on previous clinical evidence to include both donor and recipient factors, which have a key role to play in posttransplant outcome [30,31]. Regarding translational research and sample collection, our study design as a single center prospective trial may also be beneficial. Samples will be obtained according to our study protocols and by the same study members throughout the whole study period. The results of the translational part of this study may deliver novel insights into the underlying subcellular effects of HOPE in human kidney transplantation.

**Table 1:** Active prospective clinical trials on HOPE (hypothermic oxygenated machine perfusion) in kidney transplants (as assessed on clinicaltrials.gov and isrctn.com on February 27, 2019).

Trial number	Study center	Study type	Enrollment	Donor group	MP^a^ point of time
NCT03378817 (Present trial)	University Hospital Aachen, Aachen, Germany	CS^b^	15	ECD^c^-donated after brain death	End-ischemic
NCT03031067	University of Bologna, Bologna, Italy	CS	10	ECD (donated after brain or cardiac death unclear)	Unclear
NCT02621281	First Affiliated Hospital Xi’an Jiaotong University, Xi’an, Shaanxi, China	RCT^d^	400	donated after cardiac death	Immediately after retrieval
ISRCTN32967929	University of Leuven, Leuven, Belgium	RCT	210	donated after cardiac death	Immediately after retrieval
ISRCTN63852508	University Clinic Essen, Essen, Germany	RCT	262	ECD-donated after brain death	End-ischemic

^a^MP: machine perfusion.

^b^CS: cohort study.

^c^ECD: extended criteria donor.

^d^RCT: randomized controlled trial.

## Abbreviations

ATP: adenosine triphosphate
CIT: cold ischemic time
COMPARE: cold oxygenated machine preservation of aged renal donation after cardiovascular death transplants
COPE-POMP: preimplantation oxygenated hypothermic machine perfusion reconditioning after cold storage
CRF: case report file
DGF: delayed graft function
ECD: extended criteria donor
eGFR: estimated glomerular filtration rate
ELISA: enzyme-linked immunosorbent assay
ESRD: end-stage renal disease
HOPE: hypothermic oxygenated machine perfusion
HTK: histidine-tryptophan-ketoglutarate
ICH-GCP: International Conference on Harmonization-Good Clinical Practice
IRI: ischemia-reperfusion Injury
kPa: kilopascal
MPS: machine perfusion solution
NGAL: neutrophil gelatinase-associated lipocalin
O2: oxygen
pO2: partial pressure of oxygen
RWTH: Rheinisch-Westfälische Technische Hochschule
